# Editorial: The importance of cognitive practice effects in aging neuroscience

**DOI:** 10.3389/fnagi.2022.1079021

**Published:** 2022-11-18

**Authors:** William S. Kremen, Daniel A. Nation, Lars Nyberg

**Affiliations:** ^1^Department of Psychiatry and Center for Behavior Genetics of Aging, University of California San Diego, La Jolla, CA, United States; ^2^Department of Psychological Science, University of California, Irvine, Irvine, CA, United States; ^3^Department of Radiation Sciences, Umeå University, Umeå, Sweden; ^4^Department of Integrative Medical Biology, Umeå Center for Functional Brain Imaging, Umeå University, Umeå, Sweden

**Keywords:** practice effects, cognitive aging, mild cognitive impairment, Alzheimer's disease, dementia progression, cognitive intervention

Practice effects (PEs) on repeated cognitive testing is a well-known phenomenon, yet it is rarely systematically taken into account and most often simply ignored. However, failure to account for PEs can have a substantial negative impact in aging neuroscience. This Featured Resarch Topic includes 11 original research papers (cited in this editorial). We have divided them into seven non-mutually exclusive categories: (1) using level of PEs to improve prediction of progression to cognitive impairment status (Almkvist and Graff; Aschenbrenner et al.; Bender et al.; Ho and Nation; Jutten et al.; Tamburri et al.; Zheng et al.); (2) identifying predictors of reduced PEs (Bender et al.; Glisky et al.; Jutten et al.; Zheng et al.); (3) examining the magnitude of PEs associated with diagnostic severity—from cognitively unimpaired, to mild cognitive impairment (MCI) to dementia (Ho and Nation; Jutten et al.; Oravecz et al.; Tamburri et al.), or from asymptomatic mutation carriers to symptomatic mutation carriers to autosomal dominant Alzhemer's dementia (Almkvist and Graff; Aschenbrenner et al.); (4) examining PEs in normal aging (Glisky et al.); (5) adjusting cognitive scores for PEs to detect MCI earlier and characterize its progression more accurately (Sanderson-Cimino et al.); (6) using burst designs and dynamic modeling to differentiate short-term and long-term PE fluctuations and to focus on intraindividual variability (Bender et al.; Oravecz et al.; Tamburri et al.); and (7) using PEs to improve evaluation of cognitive interventions (Smith et al.).

On the surface, PEs seem simple and straightforward, i.e., they are improvements in performance on repeated testing. However, lack of improvement, and even cognitive decline, does not necessarily mean an absence of PEs. As aptly noted by some authors, it may only mean that normal aging-related or disease-related declines were still greater than the PEs (Aschenbrenner et al.; Glisky et al.; Sanderson-Cimino et al.).

All too often, we find that people are interested in which is the best method for examining PEs, frequently wanting to know if the approach being used is as good as some other approach or suggesting another approach would be preferable. Importantly, here we want to emphasize that different approaches often address entirely different issues and serve very different purposes, so trying to determine which is best is often a misguided goal. There is simply no one-size-fits-all approach. For example, several of the articles addressed the issue described in category 1 above in which the extent of PEs was used to predict individuals who would likely progress to MCI, Alzheimer's disease (AD), or other dementia (Aschenbrenner et al.; Bender et al.; Ho and Nation; Jutten et al.; Tamburri et al.; Zheng et al.). Addressing the issue described in category 5 above, Sanderson-Cimino et al. adjusted test scores for PEs based on comparison of test-naïve vs. returning participants. Doing so meant that MCI could be detected earlier and MCI progression characterized more accurately. Both sets of methods provide useful adjunctive tools for improving clinical trials and diagnostic accuracy, yet one is in no way substitutable for the other. The former approach does nothing to alter how or when the diagnosis is made. The latter does nothing to aid in predicting progression to diagnosis.

Here we note some key take-home messages regarding PEs:

1. Some studies define PEs as improvement in performance on retesting (Almkvist and Graff) or as improvement on short-term, but not long-term, retest intervals (Oravecz et al.; Tamburri et al.). However PEs are also consistently observed over intervals of a year or more (Almkvist and Graff; Bender et al.; Glisky et al.; Sanderson-Cimino et al.). Therefore, we suggest that improvements or reduced declines be referred to as PEs regardless of the size of the test-retest interval.
2. PEs make it difficult to disentangle aging-related and disease-related effects. Thus, PEs mask normal aging-related cognitive change, making it difficult to accurately characterize the course of longitudinal change. Only with matched previously untested participants at follow-up is it possible to accurately distinguish among change, effects of attrition, and PEs.
3. There is no general cognitive PE, which raises questions about the usefulness of global cognitive measures to assess PEs. It should not be assumed that the magnitude of PEs from one study would apply to another study. PEs may differ depending on:
  a. Cognitive domain
  b. Tests within a domain
  c. Age
  d. Diagnosis
  e. Duration of test-retest interval
  f. Number of repeat assessments
  g. Risk factors (e.g., AD biomarker status, brain structure, sleep, psychological wellbeing)
4. Alternate forms have been suggested as a possible way to reduce PEs (Aschenbrenner et al.). However, alternate forms make it more difficult to differentiate actual PEs from test version differences.
5. Slope of change (extent of PEs) may be a better predictor of progression to diagnosis than baseline level of function (Jutten et al.).
6. Burst designs or monthly testing are effective ways to characterize change and can be particularly useful for improved understanding of the dynamics of cognitive change, and they highlight the additional potential predictive value of within-individual variability in PEs (Bender et al.; Jutten et al.; Oravecz et al.; Tamburri et al.).
7. PEs can be usefully applied in cognitive interventions for prediction of likelihood of benefit and of transfer of training (Smith et al.).
8. Accounting for PEs by comparisons with matched previously untested participants at follow-up, results in earlier and more accurate diagnosis based on associations with reduced reversion rates of MCI and greater concordance with AD biomarkers (Sanderson-Cimino et al.).

In sum, accounting for cognitive PEs is important for accurately characterizing longitudinal change and progression to cognitive impairment status, and it is crucial to do it in a way that differentiates PEs from aging-related or disease-related change. Given the many factors that influence PEs, the magnitude of PEs cannot be expected to be comparable across studies. Incorporating PEs into clinical trials can improve participant selection efficiency and result in earlier detection of diagnostic outcomes. Such changes could also reduce study duration and staff and participant burden, which in turn, would substantially reduce costs. Only a single study in this set of papers examined PEs in the context of a cognitive intervention. Also, only a single study included matched previously untested participants at follow-up. Such matched replacements are critical for accurately distinguishing among change, the effects of attrition, and PEs. Although normative data might appear to be a solution, it provides no insight into the actual magnitude of PEs for a given age group. Given that the goals of these latter 2 studies are of great potential value, more work is called for in these areas in addition to the other areas of focus in research on cognitive PEs.

## Author contributions

WK, DN, and LN contributed to the conception and interpretation of results described in this editorial. All authors contributed to the article and approved the submitted version.

## Funding

WK was supported by grants from the United States National Institute on Aging (NIA; R01 AG050595, R01 AG876838, AG037985, AG062483, AG064955, and P01 AG055367). DN was supported by grants from the NIA (R01 AG064228, R01 AG060049, P01 AG052350, and P30 AG066519). LN was supported by a scholar grant from KAW.

## Conflict of interest

The authors declare that the research was conducted in the absence of any commercial or financial relationships that could be construed as a potential conflict of interest.

## Publisher's note

All claims expressed in this article are solely those of the authors and do not necessarily represent those of their affiliated organizations, or those of the publisher, the editors and the reviewers. Any product that may be evaluated in this article, or claim that may be made by its manufacturer, is not guaranteed or endorsed by the publisher.

